# Calcifying fibrous tumor of stomach: a rare case report of an upper gastrointestinal bleeding

**DOI:** 10.3389/fonc.2025.1512964

**Published:** 2025-04-28

**Authors:** BaoLong Ye, ZiWen Chen, JunFeng Xie, KeXing Xi, Xin Zeng, CaiLiang Zhong

**Affiliations:** ^1^ Department of Gastrointestinal and Hernia Surgery, Ganzhou Hospital-Nanfang Hospital, Southern Medical University, Ganzhou, China; ^2^ Department of General Surgery, Nanfang Hospital, Southern Medical University, Guangzhou, China; ^3^ Department of Gastrointestinal and Hernia Surgery, The Affiliated Ganzhou Hospital of Nanchang University, Ganzhou, China

**Keywords:** calcifying fibrous tumour, stomach, submucosal tumour, gastrointestinal bleeding, case report

## Abstract

**Introduction:**

Calcifying fibrous tumor (CFT) is an uncommon benign fibrous neoplastic lesion that may manifest as singular or multiple tumors and usually occurs in children or young adults. CFT originates in the muscularis propria of the stomach and is a very rare disease. Here, we report a case of gastric CFT with upper gastrointestinal bleeding.

**Case information:**

A 39-year-old male was urgently referred to our hospital with haematemesis and melena that had developed over the course of 2 hours. Enhanced abdominal CT imaging revealed a nodular lesion, measuring approximately 3.2 × 2.1 × 1.6 cm, protruding from the posterior wall of the gastric body into the gastric lumen. The lesion exhibited scattered calcifications, smooth margins, and a CT attenuation value of 50 Hounsfield units (HU). Gastroscopic ultrasonography performed in the gastroenterology department revealed a semicircular submucosal mass with signs of active bleeding. Initially, the tumor was diagnosed as a gastrointestinal stromal tumor (GIST), and surgical intervention was undertaken due to ongoing hemorrhage. Postoperative histopathological examination confirmed the diagnosis of a gastric calcifying fibrous tumor (CFT).

**Conclusion:**

CFT originating from the muscularis propria of the stomach is exceptionally rare, and the case presented here mimicked a gastric submucosal tumor (SMTs),clinicians should consider this differentia diagnosis when evaluating patients with suspected cases.

## Introduction

Calcifying fibrous tumor (CFT) is an uncommon benign fibrous neoplastic lesion that may manifest as singular or multiple tumors, usually occurring in children or young adults. The most common sites within the gastrointestinal (GI) tract are the stomach, small bowel, and colon. Most CFTs are incidentally detected during endoscopic evaluations, and the optimal treatment is local resection without lymphadenectomy (1). Gastric CFT is a very rare disease; here, we report a case of gastric CFT with upper gastrointestinal bleeding.

## Case report

A 39-year-old male was presented with haematemesis and melena that had persisted for 2 hours. The patient reported a history of intermittent epigastric pain that began approximately 1 year ago, for which no prior examination or treatment had been pursued. Neither he nor his family members had a notable medical history.

**Figure 1:**
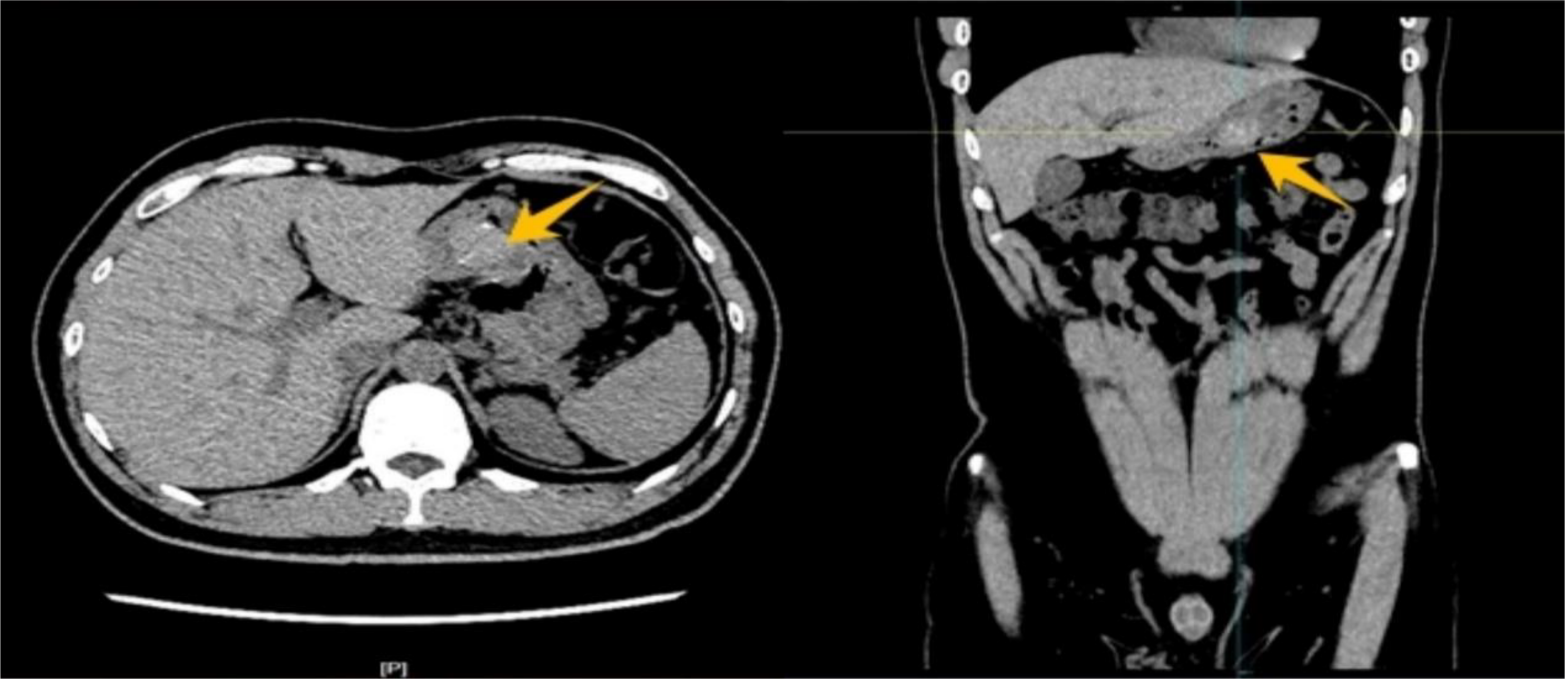
Abdominal CT imaging demonstrated a round, soft tissue density lesion on the posterior wall of the stomach with smooth margins. The plain scan revealed a CT value of approximately 71 Hounsfield units (HU), with scattered punctate calcifications within the lesion.

**Figure 2:**
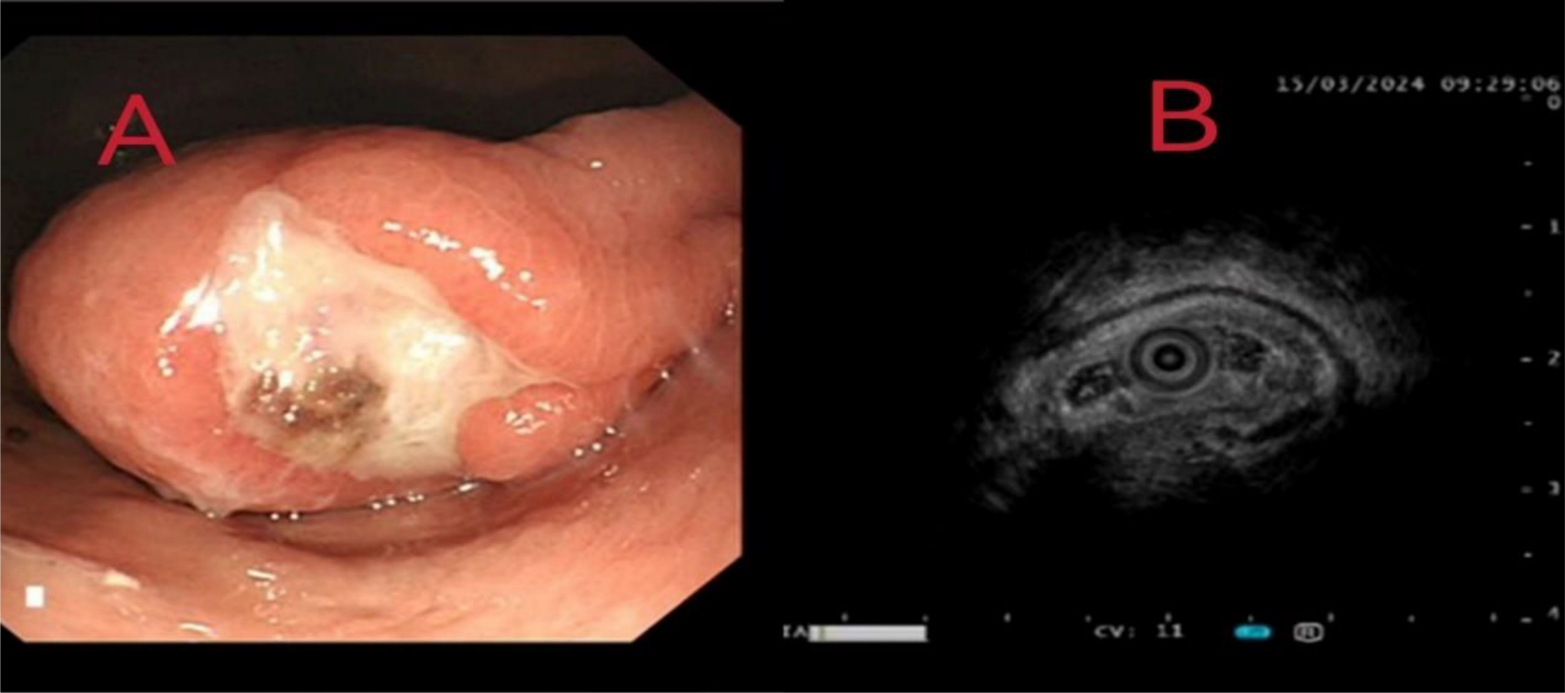
**(A)** Gastroscopic examination identified a 4.5 cm × 2.7 cm protrusion on the anterior wall of the gastric antrum. **(B)** Endoscopic ultrasonography (EUS) revealed a heterogeneous hypoechoic mass measuring approximately 4 cm × 3 cm, originating from the muscularis propria layer.

Relevant laboratory tests and diagnostic examinations were completed during hospitalization. Blood analysis revealed moderate anemia with a hemoglobin level of 79 g/L and white blood cell and platelet counts within normal ranges. The levels of serum tumor markers, including AFP, CA-199, and CEA, were all within normal limits. The remaining laboratory findings were unremarkable. Abdominal CT imaging revealed a rounded lesion with a soft-tissue density along the posterior gastric wall with well-defined margins. The unenhanced CT attenuation value was approximately 71 Hounsfield units (HU), and scattered punctate calcifications were observed within the lesion (**Figure 1**). Contrast-enhanced imaging revealed mild homogeneous enhancement. Subsequent gastroscopic examination revealed a 4.5 cm × 2.7 cm elevated lesion on the anterior wall of the gastric antrum. Endoscopic ultrasonography (EUS) revealed a heterogeneous hypoechoic mass measuring approximately 4 cm × 3 cm originating from the muscularis propria layer (**Figure 2**). Given the complex hemorrhagic nature of the tumor and the patient’s moderate anemia, surgical intervention was deferred during the initial admission period.

**Figure 3:**
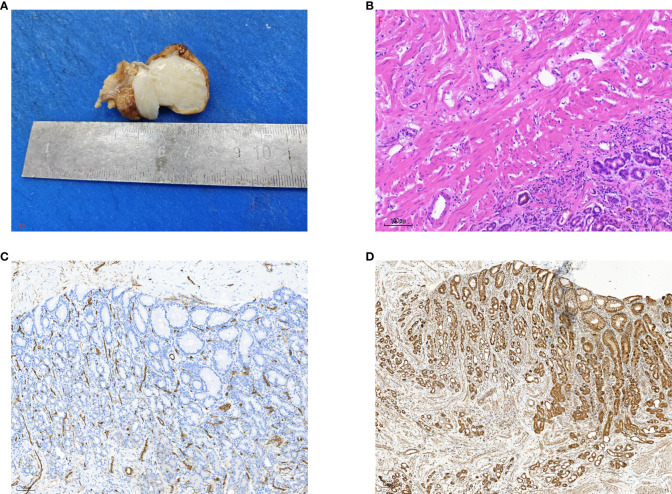
**(A)** Gross examination of the calcifying fibrous tumor showed a well-demarcated, unencapsulated, firm mass with variable calcifications on the cut surface. **(B)** Histopathological analysis revealed abundant hyalinized collagen, focal dystrophic calcifications, and a lymphoplasmacytic infiltrate (hematoxylin-eosin stain). **(C)** Immunohistochemical staining for CD34 was positive in blood vessels but negative in tumor cells. **(D)** Immunohistochemical testing demonstrated positive staining for SDHB.

Histopathological analysis of the surgical specimen revealed a greyish-white lesion on the posterior wall of the gastric fundus, measuring 3 × 2.5 × 2.0 cm microscopically. Light microscopy revealed proliferating collagenized fibrous tissue, accompanied by little calcification and localized lymphocyte infiltration. The immunohistochemistry results were as follows: CD117, scattered; DOG1, negative; CD34, positive for vascular; Desmin, negative; Ki67, 1%+; S-100, negative; SMA, negative; caldesmon, negative; and SDHB, positive (**Figure 3**).

The calcifying fibrous tumor (CFT) diagnosis for these patients was ultimately confirmed through postoperative histopathological examination. To achieve both diagnostic confirmation and therapeutic intervention, the patient underwent laparoscopic resection with complete en bloc excision of the tumor. Intraoperative exploration revealed dense adhesions among the gastric wall, gastrocolic ligament, and adjacent peritoneal surfaces. Although firm adherence to the gastric musculature was observed, the lesion was successfully dissected from the stomach wall using a meticulous surgical technique combined with harmonic scalpel application, enabling precise tissue dissection with effective hemostasis. Three-month postoperative follow-up demonstrated no evidence of tumor recurrence, which was confirmed by contrast-enhanced CT imaging.

## Discussion

Calcifying fibrous tumor (CFT) is a rare benign fibrous neoplastic lesion that can occur as solitary or multiple lesions. CFT was first reported in 1988 in two children and described as a fibrous tumor exhibiting psammomatous calcifications and lymphoplasmacytic cell infiltration in the deep soft tissue (2). In 1993, Fetsch et al. reclassified this lesion as calcifying fibrous pseudotumor (CFPT) (3), which was later designated as CFT in the 2013 World Health Organization (WHO) Classification of Soft Tissue and Bone Tumors (4). CFT predominantly affects children and adolescents, with a higher incidence in females. CFT commonly presents as a localized, slow-growing, painless mass in the subcutaneous and deep soft tissues of the limbs, trunk, axilla, groin, head, and neck. CFT has been reported in other locations, such as the pleura, mediastinum, abdomen, and pelvis, although cases involving internal organs are rare (1).

In contrast to CFTs in other soft tissues, gastric CFTs are characterized by an older age of onset, smaller tumor size, and lack of recurrence (5). Patients with gastric CFT often present with symptoms such as epigastric discomfort, hematochezia, and abdominal distension (6, 7). A meta-analysis revealed that gastric CFTs account for approximately 18.6% of all CFT cases, followed by CFTs in the small intestine (8.7%), pleura (9.9%), neck (6.2%), mesentery (5%), mediastinum (5%), and peritoneum (6.8%) (1). Gastric CFTs primarily originate in the submucosa, with involvement of the muscularis and subserosa layers occurring less frequently. On gastroscopy, gastric CFTs often appear as polypoid lesions, whereas endoscopic ultrasonography typically reveals a smooth and intact gastric mucosal surface, moderately echogenic nodules beneath the mucosa, and posterior acoustic shadowing. These features make it challenging to differentiate gastric CFTs from other submucosal tumors during endoscopic examination.

Gastric CFTs most frequently occur in the gastric body, and the pathophysiology is characterized by localized mucosal elevation. Upon sectioning, the tumor typically exhibits a well-defined submucosal nodule without a distinct capsule. The tumor has a greyish-white appearance and a medium texture and may have a gritty consistency. Studies report a calcification rate of 81% in gastrointestinal CFTs (7). Differentiating gastric CFTs from other mesenchymal tumors, such as gastrointestinal stromal tumors (GISTs) or leiomyomas, without pathological examination remains challenging. Histologically, gastric CFTs are defined by three key features: (1) abundant collagenous fibrous connective tissue with sparse spindle-shaped fibroblasts, (2) scattered calcification foci or psammomatous bodies within the collagenous stroma, and (3) interstitial infiltration of lymphocytes and plasma cells, which may form clusters, germinal centers, or, in some cases, Russell bodies (6, 7). Currently, no specific immunohistochemical markers for CFT have been identified. Fusiform cells in CFT typically show strong diffuse positivity for vimentin and focal positivity for SMA, CD34, and desmin (8). CFTs are often reported to be strongly positive for factor XIIIa and consistently negative for CD117, DOG-1, S-100, NF, Desmin, and ALK. CD34 and SMA may demonstrate focal positivity in some cases (1). The pathophysiology of gastric CFT remains poorly understood. Some studies suggest that gastric CFTs may arise as part of the inflammatory response process, potentially linked to mucosal injury in the digestive tract caused by poor dietary habits (1). Several studies have also indicated that plasma cells in this type of tumor express IgG and IgG4, suggesting a potential link between CFT and IgG4-related sclerosing disease (6, 9, 10). One study proposed that CFTs may represent the sclerotic phase of inflammatory myofibroblastic tumors (IMTs) (11), whereas another study suggested an association between CFTs and hyaline vascular Castleman disease (12). However, the underlying causes of these associations remain unclear. These hypotheses are still under debate, and further research is needed to elucidate the etiology of CFT and develop more accurate diagnostic tools. The differential diagnosis of CFT largely depends on its anatomical location. Gastric CFT needs to be distinguished from gastrointestinal stromal tumors (GISTs), leiomyomas, inflammatory myofibroblastic tumors (IMTs), schwannomas, inflammatory fibrous polyps (IFPs), and solitary fibrous tumors. GISTs are more prevalent than CFTs and have greater potential for malignant transformation. Histologically, GISTs lack calcification within the collagen fibers and hyalinized stroma, and immunohistochemistry typically indicates strong positivity for CD117 and CD34. In cases of suspected CD117-negative GIST, a panel of markers, including DOG1, CD34, S-100, SMA, CD21, ALK, and desmin, should be used to differentiate this tumor from gastric CFTs. If feasible, gene sequencing can be performed to detect c-kit or PDGFRA mutations in tumor cells. On the other hand, leiomyomas may exhibit degeneration, calcification, or ossification. Immunohistochemically, these tumors are often positive for smooth muscle actin (SMA), desmin, caldesmon, and factor XIIIa. Schwannomas exhibit features of peripheral nerve differentiation and endolymphatic characteristics similar to those of CFTs, but CFTs lack schwannomatous tissue and are consistently negative for S-100 expression (1, 13–16).

In summary, gastric calcifying fibrous tumor (CFT) is a rare benign mesenchymal neoplasm with an unclear pathophysiology. CFT primarily occurs in children and adolescents, with a relatively high incidence among females. Clinical symptoms are often nonspecific, with abdominal discomfort being the most common presentation, whereas imaging findings tend to exhibit more distinctive features. CFT should be suspected when a CT scan reveals a gastric mass with calcification; definitive diagnosis relies on pathological examination. As a benign tumor, the main treatment for gastric CFT is surgical resection. To date, no cases of recurrence or metastasis have been reported following complete tumor excision, and the overall prognosis is generally excellent.

## Data Availability

The raw data supporting the conclusions of this article will be made available by the authors, without undue reservation.
